# Immune Effector Cell–associated Hemophagocytic Syndrome (IEC-HS) as a late immune-mediated toxicity after CAR-T therapy in Diffuse Large B-Cell Lymphoma (DLBCL): a case report

**DOI:** 10.1007/s00277-026-07105-x

**Published:** 2026-06-26

**Authors:** Sofía Rincón-López, Ricardo García-Muñoz, Johelys Atencio-Matos, Olalla Pereda-Sainz, Javier Larreina-Pérez, Andrea Campeny-Najara, Pilar Herrera Pérez, Montserrat Hernández-Pérez, Jesús Feliu, Ada Esteban-Figuerola

**Affiliations:** 1 Universidad de La Rioja, Logroño, Spain; 2 Department of Hematology, Hospital Universitario San Pedro, Logroño, La Rioja 26006 Spain; 3 Cell Proliferation and Differentiation Group Oncology Area, CIBIR (Center for Biomedical Research of La Rioja), La Rioja, Spain

**Keywords:** CAR-T therapy, Diffuse Large B-Cell Lymphoma (DLBCL), Immune Effector Cell-associated Hemophagocytic Syndrome (IEC-HS), Late immune-mediated toxicity

## Abstract

Chimeric antigen receptor (CAR) T-cell therapy has dramatically improved the prognosis of patients with relapsed or refractory diffuse large B-cell lymphoma (DLBCL) [1,2]. However, immune-mediated toxicities represent a significant emerging clinical challenge, particularly those with late onset or uncommon presentations. We report the case of a 71-year-old woman with heavily pretreated DLBCL who developed immune effector cell–associated hemophagocytic syndrome (IEC-HS) with a fatal outcome six months following CAR-T infusion. This case highlights the occurrence of late -onset CAR-T-related toxicities and the potential role of CAR-T cell persistence in sustaining dysregulated immune activation.

## Introduction

Diffuse large B-cell lymphoma (DLBCL) is the most common subtype of non-Hodgkin lymphoma. Despite high initial response rates with frontline immunochemotherapy, up to 40% of patients experience relapse or refractory disease. In this setting, anti-CD19 CAR-T cell therapy has represented a paradigm shift, providing durable responses in a significant proportion of patients [1, 2]. Nevertheless, this strategy is associated with potentially severe immune-mediated toxicities, most notably cytokine release syndrome (CRS) and immune effector cell–associated neurotoxicity syndrome (ICANS), which are well-characterized and standardized according to the American Society for Transplantation and Cellular Therapy (ASTCT) criteria [3, 4]. While these complications typically occur early after infusion, late inflammatory toxicities are increasingly being recognized.

Immune effector cell–associated hemophagocytic syndrome (IEC-HS) is an uncommon but potentially fatal complication characterized by uncontrolled immune activation, hypercytokinemia, cytopenias, and multiorgan dysfunction. Its pathophysiology remains poorly understood and diagnostic overlap with infection, lymphoma progression, and classical hemophagocytic lymphohistiocytosis (HLH) frequently complicates recognition [5, 6].

We present a case of delayed ICANS followed by IEC-HS occurring six months after axicabtagene ciloleucel infusion in a patient with DLBCL, emphasizing the diagnostic challenges and the importance of considering late CAR-T–related inflammatory complications.

## Case presentation

A 71-year-old woman was diagnosed in 2023 with diffuse large B-cell lymphoma involving the adrenal glands, bone, liver, and central nervous system (CNS). Her medical history was notable for well-controlled arterial hypertension and obstructive sleep apnea.

At diagnosis, CNS involvement was considered secondary CNS lymphoma with parenchymal intramedullary (conus medullaris) and pontine infiltration. Cerebrospinal fluid (CSF) cytology and flow cytometry were negative.

The patient initially received the BONN regimen (BCNU, vincristine, methotrexate, and prednisone), which was later transitioned to R-MTX-COP (rituximab, methotrexate, cyclophosphamide, vincristine, and prednisone) due to treatment-related complications. She completed six cycles and achieved complete metabolic response. However, post-treatment MRI showed persistent T2/FLAIR hyperintensity within the right posterior periventricular white matter. The spinal cord lesion had resolved, although persistent pathological enhancement of the cauda equina was observed, consistent with residual post-treatment lesions. No repeat lumbar puncture was performed at that time.

Given the residual CNS abnormalities, consolidation with autologous hematopoietic stem cell transplantation was proposed; however, stem cell mobilization was unsuccessful.

The patient subsequently experienced disease relapse and initiated salvage therapy with R-GDP (rituximab, gemcitabine, dexamethasone, and cisplatin), completing only two cycles due to treatment-related toxicities. Follow-up PET/CT demonstrated complete metabolic response response according to Lugano 2014 criteria (Deauville score 1), and the patient was subsequently referred to the CAR-T infusion center to receive CAR-T cell therapy. R-GDP was therefore used as bridging therapy prior to CAR-T infusion. Brain and spinal MRI showed resolution of the previously described lesions, and repeat CSF flow cytometry was negative. Therefore, the patient proceeded to CAR-T infusion without radiological or cytological evidence of active CNS disease.

Pre-infusion neurological examination revealed asymmetric hypoesthesia of the right lower extremity and gait ataxia with widened base of support, which were interpreted as residual neurological sequelae from prior CNS involvement.

**Fig. 1:**
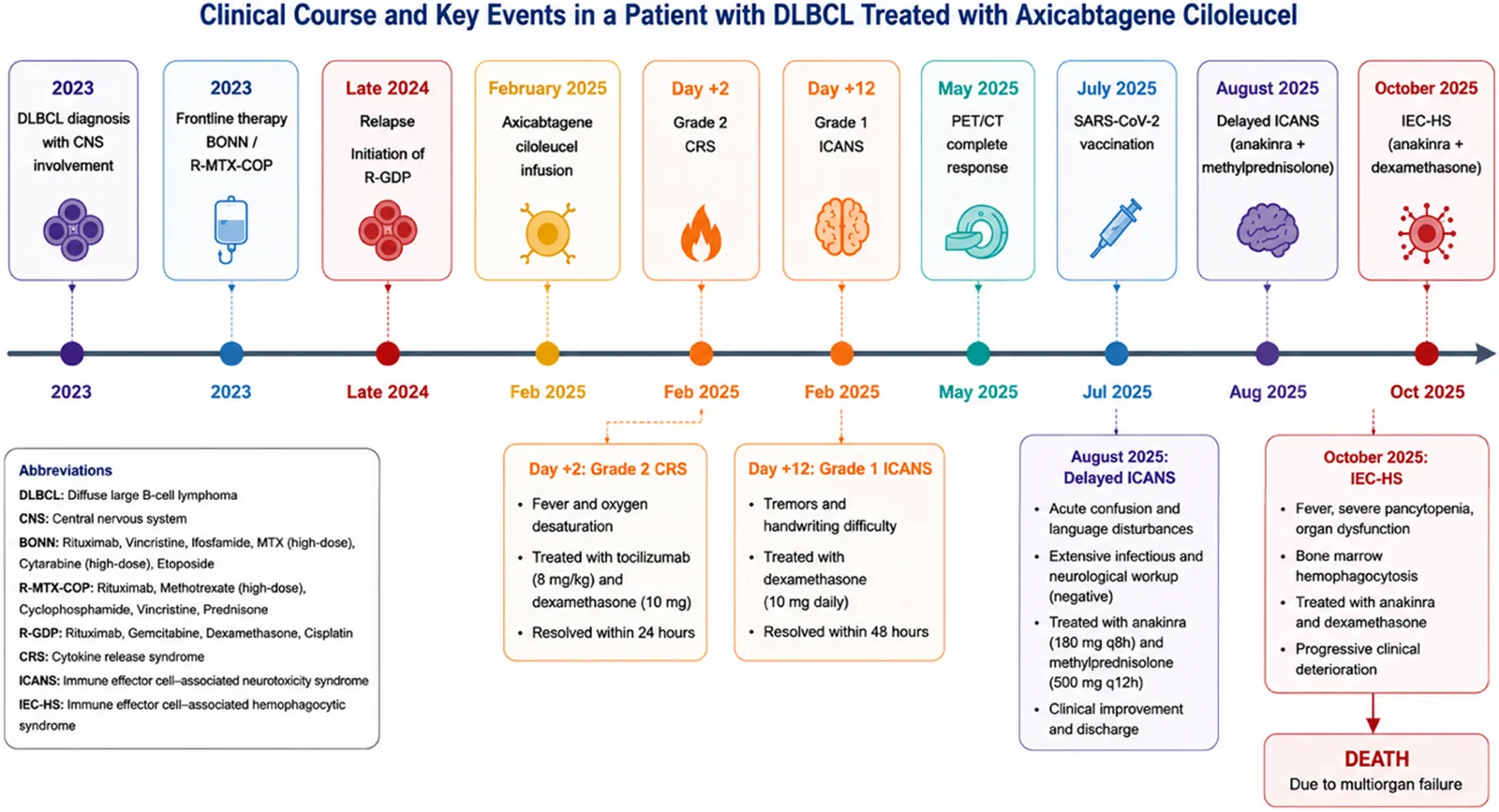
Clinical timeline of disease course and CAR-T–related toxicities

The chronological sequence of treatments, imaging findings, and immune-mediated complications is summarized in Fig. 1.

In February 2025, the patient received axicabtagene ciloleucel (axi-cel). At the time of infusion, she had no systemic manifestations of active disease and maintained an ECOG performance status of 1.

Early after infusion, the patient developed grade 2 CRS on day + 2, characterized by fever and oxygen desaturation. Symptoms resolved within 24 h after treatment with tocilizumab (8 mg/kg) and dexamethasone (10 mg). On day + 12, she developed grade 1 ICANS manifested by tremors and difficulty writing. Symptoms resolved within 48 h after dexamethasone treatment (10 mg daily).

The patient presented poor hematopoietic recovery following CAR-T therapy, with prolonged cytopenias requiring granulocyte colony-stimulating factor support, erythropoietin administration, and thrombopoietin receptor agonists. Platelet recovery remained incomplete throughout follow-up. According to the ICATH score, she had high risk for prolonged hematologic toxicity. Three months after infusion, PET/CT confirmed complete metabolic response (Deauville score 1).

Six months after CAR-T infusion, approximately one week after SARS-CoV-2 vaccination, the patient presented to the emergency department with acute confusion, language disturbances, and neurological deterioration. Blood cultures grew Staphylococcus hominis and treatment with vancomycin was initiated. An extensive infectious and neurological workup was performed during admission to the Infectious Diseases Department. Brain CT showed no intracranial complications. Brain MRI revealed only small microhemorrhages in the basal ganglia and right corona radiata, without evidence of lymphoma relapse. Video-electroencephalography demonstrated generalized bursts of nonspecific slow waves with occasional epileptiform morphology, while baseline cerebral activity remained preserved.

Two lumbar punctures were performed. CSF bacterial cultures, viral serologies, syphilis testing, toxoplasma studies, Rose Bengal test, Treponema pallidum, and Borrelia burgdorferi analyses were all negative. Flow cytometry ruled out lymphomatous infiltration. Lymphocytes represented 92% of total cellularity, of which 85% corresponded to CD4 + T lymphocytes, interpreted as a reactive increase in CD4 + T cells.

PET/CT during this admission demonstrated intense splenic uptake (SUVmax 19.7) with multiple focal lesions without splenomegaly, together with a hypermetabolic lesion in the right breast (SUVmax 4.6) and a right axillary lymph node (SUVmax 4.1). No additional disease sites were identified.

Given the suspicion of delayed CAR-T–related neurotoxicity (late ICANS), the patient was transferred to the reference infusion center, where treatment with anakinra (180 mg every 8 h) and methylprednisolone (500 mg every 12 h) was initiated. Progressive clinical improvement was observed and immunosuppressive treatment was gradually tapered over approximately 10 days.

Three weeks later, the patient was readmitted with fever (38 °C), generalized weakness, worsening neurological symptoms, and severe pancytopenia (hemoglobin 5.4 g/dL, platelets 34 × 10³/µL, neutrophils 0.5 × 10³/µL). Broad-spectrum antibiotics, intravenous immunoglobulins, transfusion support, and dexamethasone 4 mg daily were initiated.

Despite intensive management, the patient exhibited persistent fever, refractory cytopenias, and progressive neurological deterioration. A bone marrow evaluation was therefore performed to differentiate prolonged CAR-T–related aplasia from hemophagocytic syndrome.

**Fig. 2:**
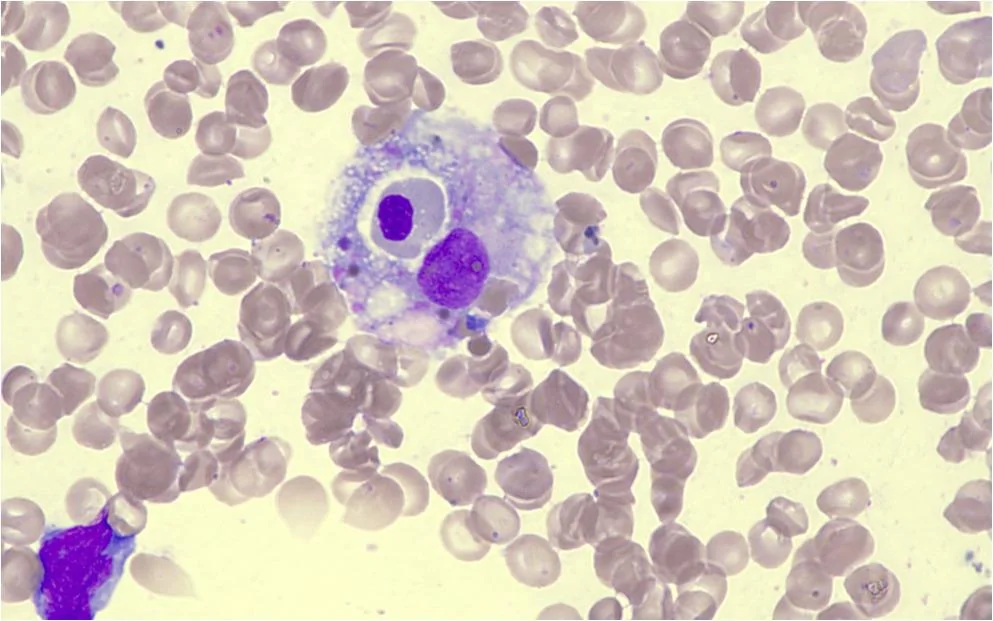
Bone marrow aspirate showing hemophagocytosis by activated macrophages. Hematoxylin and eosin (H&E) stain, original magnification (x100)

**Fig. 3:**
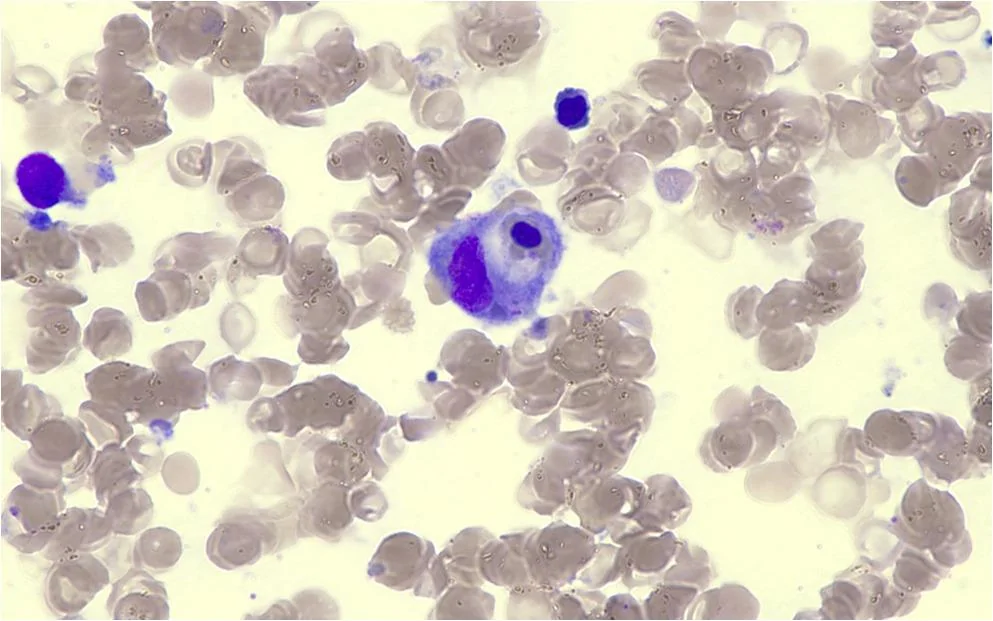
Bone marrow aspirate demonstrating hemophagocytic activity. Hematoxylin and eosin (H&E) stain, x100

**Fig. 4:**
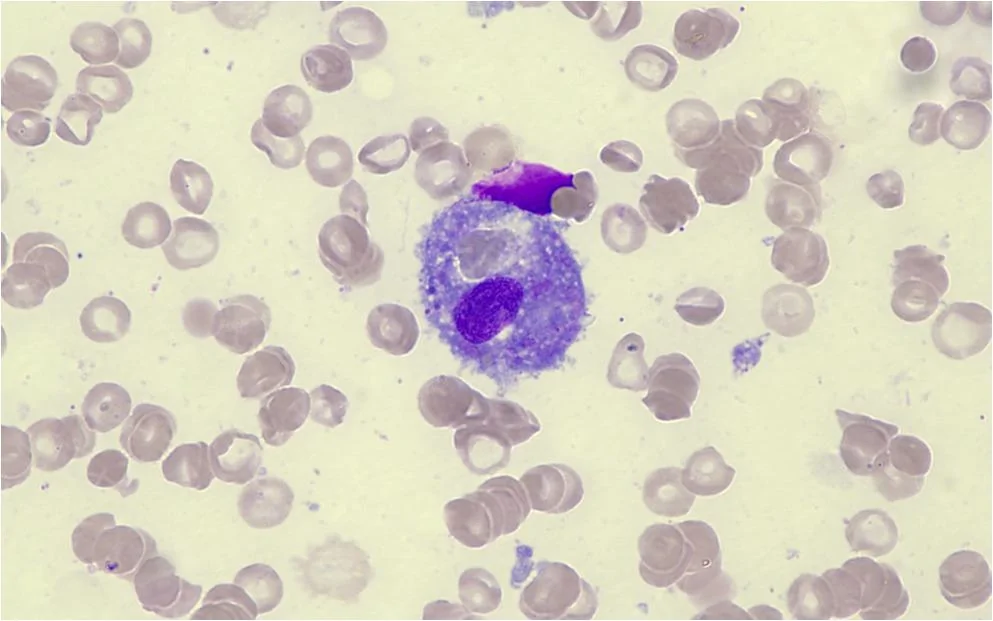
Bone marrow aspirate revealing macrophage-mediated hemophagocytosis. Hematoxylin and eosin (H&E). stain, x100

**Fig. 5:**
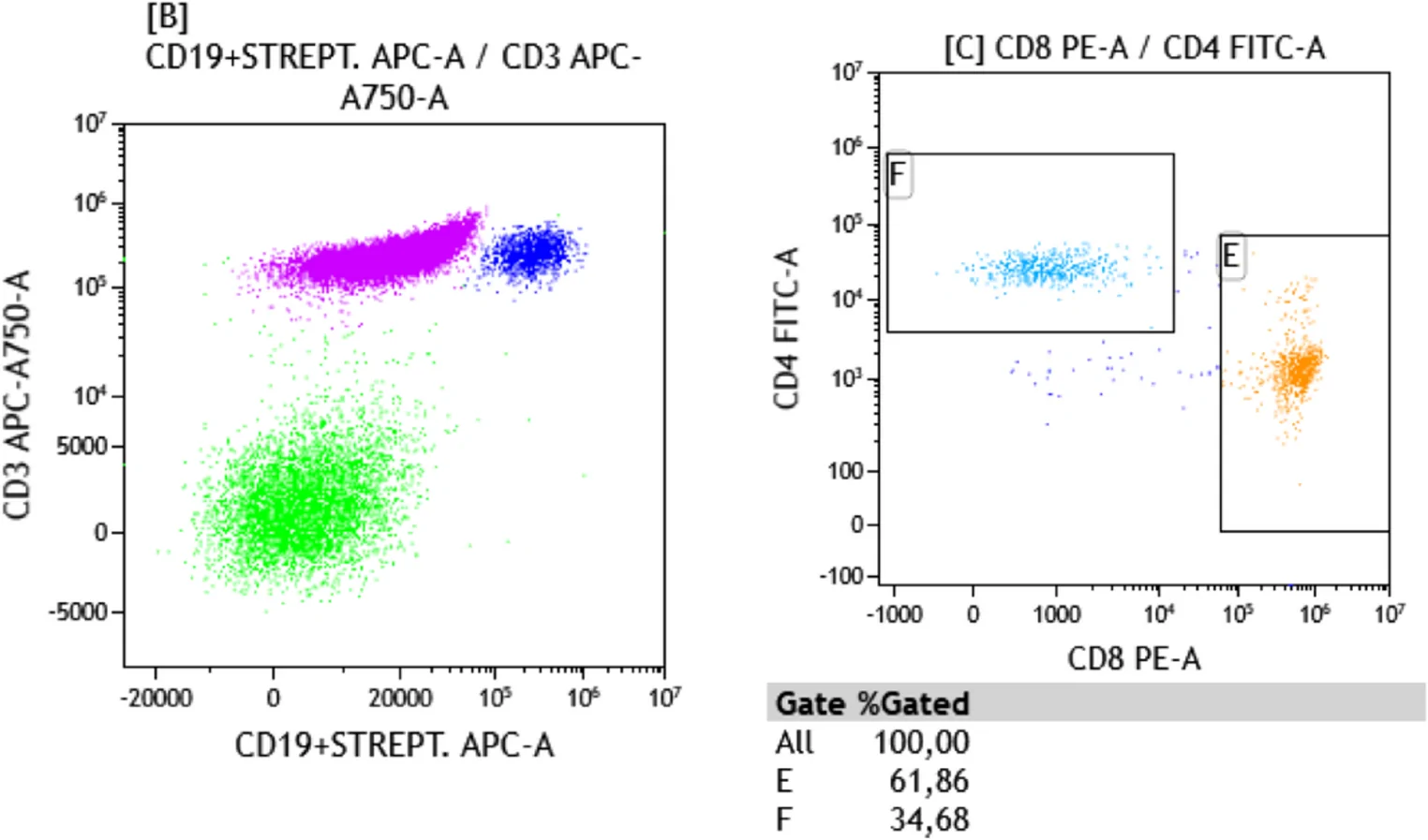
Key results from a patient treated with anti-CD19 CAR-T cells. Flow cytometric analysis of anti-CD19 CAR+/- CD19 cells (**B**), anti-CD19 CAR-T cells (**B**), and CD4/CD8 distribution (**C**). Results show our patient treated with Axicabtagene ciloleucel, demonstrating 6% CAR-T cell expansion (CD19+/CD3+). Data derived from the index patient. Flow cytometry analysis was performed on bone marrow aspirate. Cells were stained with CD45 (Krome Orange) for identification of the hematopoietic compartment and viability was assessed using 7-aminoactinomycin D (7-AAD, PC5.5). Data acquisition was performed on a Beckman Coulter Dx-Flex flow cytometer. The lymphocyte population was selected according to CD45 expression and light scatter properties. CD3 + T cells were identified using APC-A750 labeling, while CAR-positive CD19 cells were detected using streptavidin-APC

Bone marrow biopsy revealed hypocellularity, dyserythropoiesis, iron overload, and prominent hemophagocytosis **(**Figs. 2, 3 and 4**)**. Multiparametric flow cytometry identified 6% CAR-positive T cells within the lymphocyte population, with predominance of CD8 + cells (80%) over CD4 + cells (10%) **(**Fig. 5**)**.

Additional laboratory findings included ferritin 16,354 ng/mL, AST 72 U/L, ALT 43 U/L, LDH 405 U/L, triglycerides 333 mg/dL, direct bilirubin 2.3 mg/dL, PT ratio 1.46, and aPTT ratio 1.12. Fibrinogen levels remained elevated at 707 mg/dL. Renal function remained preserved with estimated glomerular filtration rate > 90 mL/min/1.73 m².

According to the diagnostic criteria proposed by Hines et al. [5], the patient fulfilled criteria for grade 2 IEC-HS (Table 1) based on prior CRS/ICANS with resolution, hyperferritinemia, persistent fever, refractory cytopenias, neurotoxicity, and bone marrow hemophagocytosis.

Anakinra (100 mg subcutaneously every 6 h) was initiated and maintained for six days. Despite immunosuppressive therapy, the patient’s clinical condition progressively deteriorated with development of multiorgan failure. After shared decision-making with the patient and her family, active treatment was withdrawn and the patient died two days later.

**Table 1:** Clinical and laboratory parameters evaluated for IEC-HS diagnosis according to *Hines et al.* [5]

Clinical / Laboratory Parameter	Result
Prior CRS/ICANS with subsequent improvement and resolution	Yes
Ferritin	16,354 ng/mL
Liver enzymes	ALT 43 U/L, AST 72 U/L
Fibrinogen	707 mg/dL
New-onset, worsening, or refractory cytopenias	Yes
Hemophagocytosis in bone marrow	Yes
LDH	405 U/L
Prolonged coagulation parameters	PT ratio 1.46, aPTT ratio 1.12
Triglycerides	333 mg/dL
Direct hyperbilirubinemia	2.3 mg/dL
New splenomegaly or renal failure	No
New or persistent fever	Yes
Pulmonary manifestations	No
Neurotoxicity	Yes

IEC-HS: immune effector cell–associated hemophagocytic syndrome; CRS: cytokine release syndrome; ICANS: immune effector cell–associated neurotoxicity syndrome; LDH: lactate dehydrogenase; PT: prothrombin time; aPTT: activated partial thromboplastin time

## Discussion

This case illustrates the broad spectrum of immune-mediated toxicities associated with CAR-T therapy, including rare but potentially lethal late manifestations. Delayed-onset ICANS occurring months after infusion is exceptional, and its pathophysiology is not fully understood. Persistent CAR-T cell activation within the CNS or a sustained inflammatory state has been proposed as a possible mechanism, suggesting a pathophysiology distinct from early-onset neurotoxicity [7, 8].

IEC-HS is an uncommon complication characterized by uncontrolled activation of the innate immune system and sustained hypercytokinemia [6, 9]. According to ASTCT criteria [6], it should be suspected in the presence of recurrent or persistent fever, severe cytopenias, hyperferritinemia, and organ dysfunction following the resolution of CRS [6, 10]. In this case, the persistence of circulating CAR-T cells with a CD8 + predominance suggests they played a key role in maintaining pathological immune activation.

An important aspect of this case is that the patient had received a SARS-CoV-2 vaccine approximately one week before symptom onset, suggesting a potential immunologic trigger. While SARS-CoV-2 vaccination is safe and recommended for most hematologic patients [11], anecdotal reports suggest it may induce transient activation of innate and adaptive immunity resulting in a “CRS-like” cytokine profile [12, 13].

Although peripheral blood CAR-T quantification was unavailable because our institution is not an infusion center, absolute lymphocyte counts increased following SARS-CoV-2 vaccination and subsequently declined after initiation of immunosuppressive therapy (Table 2). This temporal association raises the hypothesis that SARS-CoV-2 vaccination may have contributed to immune reactivation. In patients treated with cellular therapies, the inflammatory stimulus from vaccination could theoretically reactivate or expand persistent CAR-T cells or amplify systemic inflammatory circuits, precipitating late neurotoxicity or IEC-HS [14]. While cases of severe hematologic complications temporally associated with vaccination have been reported [14], establishing definitive causality remains challenging due to confounding factors such as intercurrent infection and cumulative marrow toxicity.

Management of this complication relies primarily on intensive immunosuppression with corticosteroids and cytokine blockade, particularly IL-1 inhibitors like anakinra [10, 15]. Emerging therapies such as emapalumab have shown promising activity in pediatric HLH and may represent future therapeutic alternatives for refractory IEC-HS after CAR-T therapy.

However, evidence remains limited, and the prognosis is poor, especially in cases with severe pancytopenia and neurological involvement [10].

## Conclusion

Late immune-mediated toxicities following CAR-T therapy, such as delayed ICANS and IEC-HS, are rare but devastating complications. The persistence of functional CAR-T cells may be central to their pathophysiology, potentially exacerbated by intercurrent immunologic triggers. This case underscores the need for heightened clinical vigilance and multidisciplinary management in patients with documented CAR-T cell persistence who undergo subsequent immune stimulation.

**Table 2:** Absolute lymphocyte count evolution After CAR-T therapy

Time Point	Absolute Lymphocyte Count (/µL)	CD4 + T Cells (/µL)	CD8 + T Cells (/µL)
January 2025 (Pre-infusion)	1300		
March 2025 (Infusion center)	200		
May 2025	700		
June 2025	600	81	289
July 2025 (Post-vaccination)	800	92	372
August 2025 (First admission)	900		
September 2025 (Anakinra treatment)	500 → 400		
October 2025 (Second admission)	300 → 200 → 100		

ALC: absolute lymphocyte count. The increase in lymphocyte counts after SARS-CoV-2 vaccination temporally coincided with delayed inflammatory complications and was followed by progressive decline after initiation of immunosuppressive therapy

## Data Availability

Any inquiries regarding supporting data availability of this study should be directed to the corresponding author.
